# Computable phenotypes for research using real-world data: experiences from the NIH pragmatic trials collaboratory

**DOI:** 10.1093/jamiaopen/ooag158

**Published:** 2026-08-05

**Authors:** Marisa L Conte, Judith M Schlaeger, Guilherme Del Fiol, James R Campbell, Andrea L Cheville, Keith Marsolo, Kari A Stephens, Miriam O Ezenwa, Andrew D Boyd, Juanita E Darby, Nadine S Matthie, P Michael Ho, Alice Pressman, Morgan Justice, Carol Reynolds Geary, Keturah R Faurot, Alex Fist, Karen Staman, Hulin Wu, Rachel L Richesson

**Affiliations:** Department of Learning Health Sciences, University of Michigan Medical School, Ann Arbor, MI 48109, United States; College of Nursing, University of Illinois Chicago, Chicago, IL 60612, United States; Department of Biomedical Informatics, University of Utah School of Medicine, Salt Lake City, UT 84108, United States; Department of Internal Medicine, University of Nebraska Medical Center, Omaha, NE 68198, United States; Mayo Clinic Department of Physical Medicine and Rehabilitation, Rochester, MN 55905, United States; Department of Population Health Sciences, Duke University School of Medicine, Durham, NC 27701, United States; Department of Family Medicine, University of Washington, Seattle, WA 98195, United States; University of Florida College of Nursing, Gainesville, FL 32610, United States; Department of Biomedical and Health Information Sciences, University of Illinois Chicago, Chicago, IL 60612, United States; College of Nursing, University of Illinois Chicago, Chicago, IL 60612, United States; Nell Hodgson Woodruff School of Nursing, Emory University, Atlanta, GA 30322, United States; Institute for Health Research, Kaiser Permanente Colorado, Aurora, CO 80011, United States; Kaiser Permanente Bernard J. Tyson School of Medicine, Pasadena, CA 91101, United States; Kaiser Permanente Washington Health Research Institute, Seattle, WA 98101, United States; College of Nursing, University of Nebraska Medical Center, Omaha, NE 68198, United States; Department of Physical Medicine and Rehabilitation, University of North Carolina School of Medicine, Chapel Hill, NC 27599, United States; Duke Clinical Research Institute, Durham, NC 27701, United States; Duke Clinical Research Institute, Durham, NC 27701, United States; School of Public Health, UTHealth, Houston, TX 77030, United States; Department of Learning Health Sciences, University of Michigan Medical School, Ann Arbor, MI 48109, United States

**Keywords:** electronic health records, real-world data, healthcare systems research

## Abstract

**Objectives:**

Embedded pragmatic clinical trials (ePCTs) are conducted as part of routine clinical care and therefore use data collected from real-world data sources, such as electronic health record systems and administrative claims. A common approach for using these types of data across all phases of trial conduct is to create a computable phenotype (an explicitly defined data query data, including specified data types, data codes, and logical parameters) to capture patients with a clinical condition, exposure, symptom, characteristic, treatment, or outcome of interest.

**Materials and Methods:**

The Electronic Health Record (EHR) Core Working Group of the NIH Pragmatic Trials Collaboratory captured the experiences of investigators in developing, adapting, and applying computable phenotype definitions in their studies.

**Results:**

Four case studies describe different approaches to developing and using computable phenotypes in pragmatic trials.

**Discussion:**

We recommend: (1) developing computable phenotypes as part of a team with multiple areas of expertise; (2) appropriate validation; (3) dissemination of salient details regarding phenotype creation; and (4) capturing and reporting any modifications made to phenotypes during the conduct of the trial.

**Conclusion:**

A range of issues and decisions influence how computable phenotypes are developed and used in pragmatic clinical trials. Multidisciplinary teams that understand the context of the data, including the reason the data are collected and potential sources of bias, are best suited for the development and validation of phenotypes for ePCTs.

## Introduction

Pragmatic clinical trials embedded in healthcare systems are designed to inform decision-makers about the benefits, burdens, and risks of a biomedical or behavioral health intervention.^1^ Most embedded pragmatic clinical trials (ePCTs) use data collected during routine care for clinical or administrative purposes, such as data from electronic health records (EHRs) and related claims, and administrative or clinical information systems. An approach for standardizing the use of real-world data across all phases of a trial is to create a computable phenotype (ie, a query of data, including specified codes and logical parameters). Computable phenotypes typically include clinical concept representations and programming logic^2^ and are used to capture characteristics of interest, such as patients with a clinical condition, exposure, symptom, characteristic, treatment, or outcome of interest.

However, the use of real-world data for research purposes poses many challenges.^3^^,^^4^ Data from individual sites reflect differences in local documentation practices and data collection systems, despite nationally recognized and widely adopted terminology standards, for example Current Procedural Terminology (CPT), International Classification of Diseases (ICD), Logical Observation Identifiers Names and Codes (LOINC), RxNorm, and the Systematized Nomenclature for Medicine (SNOMED CT).^5^^,^^6^ Variation in documentation practices also occurs at the clinician level,^7^^,^^8^ due to user preferences and differences in EHR designs.^9^ Despite the development of repositories,^10^^,^^11^ libraries,^12^^,^^13^ and frameworks^2^^,^^14^ designed to support the curation of phenotypes and promote their retrieval and reuse, the actual reuse of existing computable phenotypes—especially in ePCTs—remains elusive.

Thus, we sought to create a collection of examples that illustrate tangible issues and decisions that influence how computable phenotypes are developed, refined, and used in current, real-world ePCTs.

## Methods

The NIH Pragmatic Trials Collaboratory has supported the design and execution of 35 ePCTs that address issues of major public health importance and engage healthcare delivery systems in research partnerships.^15^ To support broader awareness of challenges and solutions to developing and using computable phenotypes for ePCTs, members of the Collaboratory’s EHR Core shared their experiences in creating and using computable phenotypes, collated and discussed a selection of examples, and developed recommendations. These 4 trials were selected because they collectively illustrate distinct aspects of computable phenotype development and use across ePCT conduct: participant identification under imperfect data quality (BeatPain Utah), safety outcome monitoring requiring iterative multidisciplinary validation (BEST ICU), phenotype construction for a clinically complex condition with coding variability (GRACE), and fully automated EHR-embedded enrollment using nonstandard vendor data (NOHARM).

### Case studies and lessons learned

1. BeatPain Utah^16^: identifying chronic pain patients as potential participants (Table 1)
**Study goal:** Evaluate the impact of telehealth-based management of chronic low back pain in federally qualified health centers (FQHCs).
**Setting:** Two EHR vendors across 11 independent community FQHCs in Utah. EHR data from all FQHCs are exchanged and normalized via daily automated data interfaces with a centralized data warehouse managed by the Utah Primary Care Association.
**Reason for phenotype:** To identify patients with a provider encounter who had a diagnosis of chronic low back pain.
**Composition of phenotype**: ICD-10 encounter diagnoses, encounter locations, and CPT procedure codes.
**Challenges:** (1) not all data required to assess exclusion criteria were available in the EHR as structured data; and (2) there were inaccuracies in encounter diagnosis coding (eg, acute back pain coded as chronic).
**Solution:** Used a computable phenotype to screen for potential enrollment. Potentially eligible patients identified by the computable phenotype received automated, bidirectional text messaging outreach offering enrollment in the study. Patients who replied “YES” were contacted by a physical therapist who verified eligibility, completed enrollment, and administered study interventions.
**Lesson learned:** The computable phenotype was best used as part of a multistep screening process, with physical therapists confirming eligibility over the phone, as inaccuracies in the encounter diagnoses data were found.
**Recommendation for trialists:** Researchers conducting multi-site trials should understand the meaning and limitations of the data for each participating site. Variance in data availability and quality across sites may lead to subtle changes in the protocol requiring additional steps for identifying participants.
2. BEST ICU: leveraging standards and diverse expertise in critical care
**Study goal:** Implement and evaluate behavioral interventions to foster ABCDEF bundle^17^ adoption, thereby improving the clinical outcomes of critically ill adults who require treatment with invasive mechanical ventilation.
**Setting**: 12 intensive care units (ICU) at 3 hospital systems that have implemented a common data model as part of their participation in the Patient Centered Outcomes Research Network (PCORnet).^18^
**Reason for phenotype:** At the direction of the study Data Safety Monitoring Board, a computable phenotype was developed to identify the appearance of new cardiac arrhythmias in patients receiving the ABCDEF bundle. The computable phenotype targets surrogate events reflecting the occurrence of a novel arrhythmia, specifically cardioversion procedures and/or the ordering of newly prescribed antiarrhythmic medications.
**Composition of phenotype:** Cardioversion procedures were reliably documented as billed procedures with CPT4 code 92960. Additionally, medication administration logs populated in the EHR as a part of the barcode medication administration process during inpatient stays and the medication prescriptions in outpatient care were reliable datasets for comparing medications administered during the ICU stay with the patient’s treatment history.
**Challenge:** To examine the validity of the computable phenotype (CP) for identification of new arrhythmias, the study team sampled the enrolled patient population and reviewed the EHR for newly diagnosed arrhythmias. In the sample, the team found that 9% of patients experienced such an event. The CP extract identified 42% of the affected patients and correctly classified 76% of the unaffected patients. Unfortunately, due to the low prevalence of arrhythmia events, the CP only correctly reported affected patients 1 time in 6. The safety team agreed that this performance was not acceptable.
**Solution:** Analyzing the reviewed cases, the study team determined that patients were often admitted to the ICU because of a hospital-acquired arrhythmia, and the investigators concluded that they should ignore events within 24 hours of ICU admission as they represent arrhythmias unrelated to the study intervention. The validation activities also identified patients administered intravenous lidocaine—used for ventricular arrhythmias—who were being treated for propofol-induced pain during induction of anesthesia. From this observation, investigators proposed excluding cases of intravenous lidocaine use by an anesthesiologist. Finally, the team discovered that patients receiving oral dosing of amiodarone were often being prepared for surgery but had experienced no arrhythmia, so they refined the computable phenotype query to include only intravenous amiodarone administration. The team has modified the computable phenotype and is now in the second round of data accrual and validation, with results pending.
**Challenge:** EHR data can be used to identify and describe events but do not necessarily explain the administrative, business, or clinical context. For example, examination of the variable “Duration of stay in the intensive care unit” during validation found that bed occupancy data overestimated the duration of treatment time by the critical care team because some patients with transfer orders remained in the ICU beds until an acute care bed became available.
**Solution:** Another class of EHR data (transfer orders) was queried and added to the computable phenotype to identify the correct date and time for the end of critical care treatment.
**Lesson learned:** The study’s data management team was intentionally organized to leverage the expertise of clinicians, EHR analytics specialists, EHR data extraction specialists (SQL programmers), and vendor consultants. Each member of this multidisciplinary team was needed to understand the context and interpretation of the data to develop and validate the computable phenotype.
**Recommendations for trialists:** Partnerships between local data experts, clinical experts, and the research study team are essential. Even in collaborations or research networks with tremendous investment in data and informatics, these resources, and the data they process, are not completely “research ready” for translation to a new research question or study.
3. GRACE: identifying a complex disease presentation with chronic pain^19^
**Study goal:** To determine the effectiveness of guided relaxation and acupuncture, compared to usual care, for reducing chronic pain and opioid use among adult patients with sickle cell disease (SCD).^19^
**Setting:** This trial initially involved 3 large healthcare systems and expanded to include 5 sites to meet recruitment goals.
**Reason for phenotype:** To screen patients for a diagnosis of an SCD as the first step for determining study eligibility. Enrollment criteria were: (1) relevant ICD-10 diagnosis codes; and (2) minimum scores on 2 validated pain scales.
**Composition of phenotype:** ICD-10 diagnosis codes
**Challenges:** While several codes capture SCD with acute pain, there is no single ICD-10 code to identify SCD with chronic pain. Nuances in clinical presentation of SCD include a myriad of sequelae such as acute chest syndrome, stroke, retinopathies, priapism, nephropathies, avascular necrosis, and leg ulcers.^20^ Additionally, the assignment of ICD-10 diagnosis codes may vary based on a several factors, including region, practice patterns, unit of time, and case complexity.
**Solution:** The study team worked with SCD physicians at the study sites to identify relevant diagnostic codes. The final code list included 10 ICD-10 codes that are associated with SCD and common sequelae. This method of accounting for both the nuanced presentation and practice-based coding variability increased the computable phenotype’s ability to identify potential participants.
**Recommendation for trialists:** Clinicians at study sites can share valuable information about processes, such as coding practices that may influence data collection, and local data experts have a wealth of knowledge about how disparate data elements can be combined to characterize a condition or characteristic of interest.
4. NOHARM: challenges of nonstandard data^21^
**Study goal:** This study evaluated the impact of a multicomponent EHR-embedded intervention on the uptake of nonpharmacologic modalities (eg, acupressure, mindfulness) for postoperative pain, and on patient-centered outcomes and opioid use. Episodes of care for qualifying surgeries served as the unit of analysis. These were automatically identified by an EHR algorithm that reviewed initial EHR surgery orders for a digital phenotype. Thereafter, given waiver of informed consent, enrollment occurred automatically.
**Setting:** Four large integrated healthcare systems. A unified EHR supports all participating sites and practices, and the sources of information and standards for data entry were consistent at all trial sites.
**Reason for phenotype:** Screening for eligibility and study enrollment. Inclusion criteria included surgical procedure codes, provider identification, clinical encounter type and location, and encounter-associated surgical departments, divisions, and clinics. The computable phenotypes were built into the EHR as patient enrollment algorithms that automatically evaluated newly placed surgical orders within the EHR.
**Challenge:** Inclusion criteria for qualifying surgeries were not consistently represented in the EHR, most notably the type of surgery and surgeons performing the procedure. These challenges were due to insufficient standards and semantic representation of important concepts. For example, defining characteristics of surgical procedures and intraoperative procedure modifications are not consistently represented within discrete fields of the EHR. Vendor practices also complicated computable phenotype development. For example, Epic uses a nonstandard and less granular coding system to document surgical procedures. Some issues were not noticed until after trial completion and data abstraction. For example, issues arose when multiple operating room procedure (ORP) codes were assigned to a specific surgery or procedure, when conversions from one operation to another occurred intraoperatively, and when multiple providers from differing departments co-performed a procedure, in either a pre-planned or emergent fashion.
**Solution:** Use of the Epic ORP surgical and procedural codes required aggregation with other data types, including site, surgeon, department, and provider characteristics, to achieve an acceptable level of accuracy in identifying qualifying procedures.
**Lesson learned:** Validation of computable phenotypes is necessary^22^ but the appropriate process may vary by study type, ranging from face validity—for example, in the case of trials that are utilizing the phenotype as one step in eligibility screening—to more formal and rigorous validation for phenotypes used for study design elements such as eligibility determination and study outcomes.^23^
**Recommendation for trialists:** Be prepared to iteratively develop the phenotypes to work with local data and continuously validate phenotype performance.

**Table 1.** Participating NIH pragmatic trials collaboratory trials and lessons learned.

Trial	Study goal	Study setting and population	EHR use	Lesson learned
**BeatPain Utah** Nonpharmacologic Pain Management in Federally Qualified Health Center Primary Care Clinics NCT04923334	Improve pain management and reduce reliance on opioids for patients with chronic back pain in federally qualified health centers in Utah	Adults with chronic back pain in federally qualified health centers in Utah	Determine eligibility, outcomes	The computable phenotype was best used as a part of a multi-step screening process, with physical therapists confirming eligibility over the phone, as there were inaccuracies in the encounter diagnoses data in the EHR.
**BEST-ICU** Behavioral Economic and Staffing Strategies to Increase Adoption of the ABCDEF Bundle in the ICU NCT06184945	Build and evaluate behavioral economic and implementation science theory-based interventions fostering ABCDEF bundle adoption and improving the clinical outcomes of critically ill adults	Critically ill adults on mechanical ventilation in 12 intensive care units in 3 hospitals	Part of intervention delivery, outcomes	The development of a phenotype is best done with the advice of a team that includes clinicians, EHR analytics specialists, EHR data extraction specialists (SQL programmers) and vendor consultants and with diverse expertise. EHR data reflects events but does not necessarily explain the context.
**GRACE** Hybrid Effectiveness-Implementation Trial of Guided Relaxation and Acupuncture for Chronic Sickle Cell Disease Pain NCT04906447	Determine the effectiveness of guided relaxation and acupuncture in comparison to usual care in reducing chronic pain and opioid use among patients with sickle cell disease	Adults with sickle cell disease and chronic pain in 5 large healthcare systems	Determine eligibility, outcomes	Work with a multidisciplinary team to identify all codes that could be attributable to a condition (cast a wide net), then confirm via follow-up. The addition of in-person recruitment may also be necessary.
**NOHARM** Nonpharmacologic Options in Postoperative Hospital-based and Rehabilitation Pain Management NCT04570371	Evaluate the feasibility of EHR-embedded patient- and clinician-facing decision support for nonpharmacologic pain care after surgery	Approximately 54 000 postoperative patients in 22 practice clusters in 4 large integrated healthcare systems	Determine eligibility; part of intervention delivery; outcomes	Be aware of data that do not conform to conventions and include other data types to improve algorithm performance.

## Discussion

The 4 case studies highlight successes and challenges in computable phenotype development and use in ePCTs and demonstrate how both pragmatic and scientific considerations inform the development and use of computable phenotypes. For most NIH Collaboratory trials, computable phenotypes were used as part of a multistep screening process, which often involved human review and iteration. To enhance transparency in the development of computable phenotypes, ePCT investigators should provide salient details about the creation and use of phenotypes in their trials, including: the purpose of the phenotype, the data types used and their sources for data, how the phenotype was validated, and any modifications made to accommodate differences across study sites. Details about the creation and use of computable phenotypes increase the interpretability of the trial’s results and can also be used to assess the comparability of data across sites and trials to serve as exemplars for future researchers. We recommend that decisions about the design and execution of computable phenotypes be shared as part of trial reporting. Understanding these decisions facilitates a greater understanding of the study population and is important to a variety of audiences, particularly researchers who wish to replicate a study, reuse existing phenotypes in part or in whole, or design and adopt EHR-integrated interventions.

### Limitations

We present only a limited set of experiences from the NIH Pragmatic Trials Collaboratory, and there may be additional lessons learned across a range of different projects.

## Conclusion

Together, our case studies advance understanding of how real-world data challenges manifest differently depending on the intended purpose of the developed phenotype, the clinical context, and the infrastructure of the participating sites. A key insight across all 4 examples is that computable phenotypes rarely function as static, fully-specified queries; rather, they require iterative refinement informed by clinical expertise, local data knowledge, and ongoing validation. These cases also demonstrate that the degree of rigor required for validation is not uniform—it scales with the phenotype’s role in the trial, from a first-pass eligibility screen to an automated enrollment trigger to a safety monitoring tool. By documenting these varied experiences, this collection provides a practical reference for future ePCT investigators navigating similar challenges and reinforces the value of transparency in reporting how phenotypes are developed, modified, and applied throughout a trial.

As our experience with real-world data grows, so does our understanding that their use must be coupled with other processes and validation procedures. While electronic data from a multitude of sources can be used to characterize patients and their experience, human oversight is still needed for verification. Multidisciplinary teams that understand the context of the data, including the reason the data are collected and potential sources of bias, are best suited for the development and validation of phenotypes for ePCTs.

## Data Availability

Data are available from the corresponding author upon request.
